# Feasibility of simultaneous PET-MR perfusion using a novel cardiac perfusion phantom

**DOI:** 10.1186/s41824-017-0008-9

**Published:** 2017-10-12

**Authors:** Jim O’Doherty, Eva Sammut, Paul Schleyer, James Stirling, Muhummad Sohaib Nazir, Paul K. Marsden, Amedeo Chiribiri

**Affiliations:** 1 Division of Imaging Sciences and Biomedical Engineering, PET Imaging Centre, King’s College London, St. Thomas’ Hospital, 1st Floor Lambeth Wing, St Thomas’ Hospital, London, SE1 7EH UK; 2 Division of Imaging Sciences, King’s College London, Wellcome Trust/EPSRC Medical Engineering Centre, St. Thomas’ Hospital, London, UK; 3 Bristol Heart Institute, Bristol, UK; 4 Siemens Healthcare Limited, Frimley, Camberley, UK; 5 Department of Cardiology, Guy’s and St Thomas’ NHS Foundation Trust, London, UK

**Keywords:** PET-MR, Cardiology, Perfusion, Flow

## Abstract

**Background:**

PET-MR scanners are beginning to be employed for quantitative myocardial perfusion imaging. In order to examine simultaneous perfusion calculations, this work describes a feasibility study of simultaneous PET-MR of gadolinium-based contrast agent (GBCA) and PET radiotracer in a novel cardiac perfusion phantom.

**Results:**

[^18^F]F^−^ and GBCA were injected simultaneously into a cardiac phantom using a range of ground-truth myocardial perfusion rates of 1 to 5 ml/g/min. PET quantification of *K*_*1*_ (ml/g/min) was performed using a single tissue compartment model. MR perfusion was calculated using a model-independent signal deconvolution technique. PET and MR signal traces from the phantom aorta and myocardial sections show true simultaneous PET and MR arterial input functions (AIF) and myocardial uptake respectively at each perfusion rate. Calculation of perfusion parameters showed both *K*_*1*_ and *h(t = 0)* (PET and MR perfusion parameters respectively) to be linearly related with the ground truth perfusion rate (*P*_*T*_), and also linearly related to each other (R^2^ = 0.99). The highest difference in perfusion values between *K*_*1*_ and *P*_*T*_ was 16% at 1 ml/g/min, and the mean difference for all other perfusion rates was <3%.

**Conclusions:**

The perfusion phantom allows accurate and reproducible simulation of the myocardial kinetics for simultaneous PET-MR imaging, and may find use in protocol design and development of PET-MR based quantification techniques and direct comparison of quantification of the two modalities.

## Background

Cardiac magnetic resonance (CMR) plays an increasing role in the diagnosis and stratification of patients with suspected coronary artery disease (CAD) justified by its high spatial resolution, tissue contrast and the ability to provide reproducible quantitative data on parameters such as left ventricular volumes and mass. CMR is also increasingly used to assess inducible ischaemia (Fihn et al., 2012; Task Force et al., 2013). In carefully controlled situations, CMR techniques have been shown to also provide absolute quantitative measurements of myocardial blood flow (MBF) and myocardial flow reserve (MFR) (Jerosch-Herold, 2010).

Positron emission tomography-computed tomography (PET) imaging is a highly accurate method for assessment of obstructive coronary artery disease (CAD), with a sensitivity and specificity of approximately 90% (Di Carli et al., 2007) and is considered the reference method for non-invasive quantification of myocardial perfusion (Bengel et al., 2009). Dynamic PET imaging can be performed using short-lived metabolized tracers (e.g. [^82^Rb]Cl, [^13^N]NH_3_) or freely-diffusible tracers (e.g. ^15^O–H_2_O) for quantification of absolute MBF and MFR.

The recent introduction of simultaneous hybrid PET-MR systems for combined molecular and functional imaging could be of great use in terms of understanding underlying cardiac pathophysiology and improving cross-modality validation. Multiple images comprising structural and functional information of the same tissue in the same physiological state can be acquired simultaneously. The combination of PET and MR acquisitions can provide further benefits in cardiac imaging such as real-time motion correction (Petibon et al., 2013), reduced patient scan time compared to independent CMR and PET-CT scans (Ratib & Nkoulou, 2014), and a reduction in exposure to ionizing radiation (Ratib et al., 2013).

Due to the demanding technical requirements of first-pass perfusion imaging, the use of simultaneous PET-MR systems for quantitative cardiac imaging is only just emerging. Sequential CMR and PET perfusion measurements in a on the same day have shown that physiological variations in the time between studies (i.e. hemodynamic conditions) are a major factor (Morton et al., 2012). Recent work has performed simultaneous PET-MR in [^18^F]FDG cases to examine cardiac viability (Nensa & Schlosser, 2014), cardiac sarcoma (Nensa et al., 2015) and active inflammation imaging of cardiac sarcoidosis (Schneider et al., 2014). MFR determined from [^15^O]H_2_0 PET from both PET-MR and PET-CT systems has been compared from 10 patients, detailing a high intra-class correlation coefficient of 0.98 (Kero et al., 2017). Another group studied the feasibility of acquiring MR and PET perfusion profiles simultaneously using dynamic contrast enhancement MR (DCE-MR) and [^13^N]NH_3_ PET for 10 patients, showing a correlation of R^2^ = 0.67 for rest and stress MBF and R^2^ = 0.48 for MFR (Zhang et al., 2013). A major confounding factor in the correlation between PET and MR perfusion comparison involves the difference in the tracer mechanism. Gadolinium based contrast agents (GBCA) do not undergo any intracellular processes, remaining distributed only within the extracellular space, whereas PET radiotracers typically enter and exit the myocyte. Thus there is also a lack of similarities between approaches to quantify perfusion on CMR and PET techniques such as modeling assumptions, fitting methods and parameter constraints (Gerber, 2012). Also of note in simultaneous imaging is the potential effects of contrast agent on the MR-based map for attenuation correction of PET sinograms (Rischpler et al., 2013; Rischpler et al., 2015).

There is thus room to improve correlation between PET and CMR perfusion quantification techniques, and a physiologically validated phantom with the capability of simultaneous PET-MR acquisition is likely to add to the growing body of knowledge. Our perfusion phantom has previously been validated to provide data suitable for quantitative analysis (Zarinabad et al., 2012) and has been employed in MR (Chiribiri et al., 2013) and CT quantification (Otton et al., 2013). Our work here follows on from our first investigations of simultaneous PET-MR phantom acquisitions using simultaneous injections of radiotracer and GBCA (O'Doherty et al., 2016). We aimed to investigate if perfusion estimates calculated independently via PET and MR techniques are related.

## Material and Methods

### Phantom

**Fig. 1:**
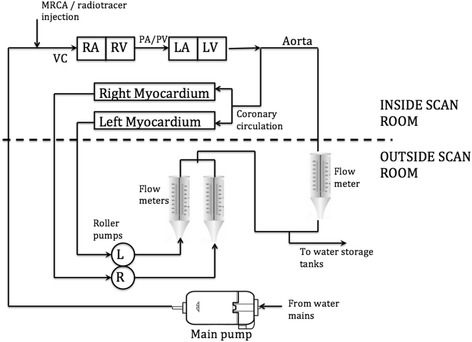
Basic schematic representation of the phantom showing the control unit outside the scan room and the phantom components inside the PET-MR scanner. VC = vena cava, PA/PV-pulmonary artery/vein, RA/LA = right/left atrium, RV/LV-right/left ventricle. Image modified from Chiribiri et al. (Chiribiri et al., 2013)

We used an in-house designed and built myocardial perfusion phantom, which has previously been described in detail (Chiribiri et al., 2013). Briefly, water is pumped through an MR-safe myocardial perfusion phantom placed in the scanner. The phantom is representative of the large thoracic vessels and of the heart of a 60 kg subject. It is composed of four cardiac chambers (120 ml each) and associated thoracic vessels (aorta, pulmonary artery, pulmonary vein, vena cava). A schematic representation detailing the phantom itself and supporting precision pumping and monitoring mechanisms is shown in Fig. 1. Myocardial perfusion is controlled in real time by flow meters continuously sampling the flow rate by means of high-precision digital flow meters (Atrato, Titan, Sherborne, United Kingdom) and providing re-adjustment of the speed of rotation of roller pumps through a feedback mechanism. Perfusion values were obtained by means of measurements of the distribution volume for the radioactive tracer and for the GBCA, and dividing the flow rate by this value. All pump controls and flow/perfusion rates are handled remotely from a custom-written LabVIEW application (LabVIEW Professional Development System 2014, National Instruments, Austin TX, USA) running on dedicated workstation and remotely controlled using an iPad application (Dashboard for LabVIEW, National Instruments, Austin TX, USA). As no radiotracer or GBCA re-entered the system after injection, we utilized a non-recirculating model in order to study first-pass myocardial perfusion measurements.

### Scanning parameters

We performed PET-MR imaging on a 3 T Siemens Biograph mMR scanner (Siemens Healthcare GmbH, Erlangen, Germany). The MR sequence consisted of a clinically utilized imaging protocol, namely a 2D TurboFLASH saturation recovery gradient echo sequence (TE = 1 ms, TR = 164 ms, Flip angle = 10^o^, slice thickness = 6 mm, pixel spacing = 1.875 mm, matrix size 144 × 192 voxels, with temporal resolution of 1 image per cardiac beat). MR data were acquired in a single transverse plane identified by markings on the phantom, the locations of which correspond to a known dispersion volume for the GBCA and radiotracer. Cardiac output flow rate was set to 3 l/min, with true myocardial perfusion rates (hereon denoted *P*_*T*_) set to 1, 2, 3, 4 and 5 ml/g/min. A previously validated dual-bolus protocol was used for GBCA injection, with a pre-bolus of 0.001 mmol/kg of GBCA injected before a main bolus of 0.01 mmol/kg (Ishida et al., 2011). A minimum pause of 30 s was allowed between the pre-bolus and the main bolus of GBCA to ensure return of signal in the vascular and myocardial compartments to baseline values.

3D PET data were acquired in a single list-mode file and re-binned into short frames during the peak influx and washout phases (60 × 3 s) and longer frames towards the end of the washout phase (12 × 15 s). PET image frames were reconstructed using the standard manufacturer-issued filtered back-projection (FBP) algorithm available on the scanner (344 × 344 matrix, Gaussian smoothing filter of 4 mm. Resulting PET voxel sizes were 2.086 mm × 2.086 mm × 2.031 mm. Attenuation correction of PET data was provided by the standard dual-point VIBE T1-weighted Dixon sequence available on the mMR scanner front end (Martinez-Moller et al., 2009). Total attenuation of the phantom is low as there is no attenuating material surrounding the phantom.

A mean injected activity of 207.8 ± 9 MBq was injected in order to exclude potential confounding dead-time effects in the PET detectors which has been shown to occur in clinical situations (O'Doherty et al., 2014; Renaud et al., 2016). After preloading of [^18^F]F^−^ into the tubing, the main bolus of GBCA (Gadovist®, Bayer HealthCare, Berlin, Germany) and [^18^F]F^−^ were injected simultaneously via a contrast injection system (Spectris Solaris, Bayer AG, Leverkusen, Germany) through the vena cava tubing of the phantom (Fig. 1). Simultaneous dynamic PET-MR imaging was performed for a total of 300 s. A single simultaneous PET-MR acquisition was performed at each *P*_*T*_ step, and each step was repeated for an estimate of repeatability of the phantom. After each scan, water was pumped through the myocardial compartments of the phantom for a minimum of 60 s between experiments to ensure a complete washout of GBCA and radiotracer before the next scan.

### Image analysis

Dynamic PET images were analyzed in PMOD v 3.7 (PMOD Technologies, Zurich, Switzerland) to produce time-activity curves (TACs). 2D MR images were analyzed in OsiriX (OsiriX 64-bit, version 8.0.2, Pixmeo SARL, Geneva, Switzerland) to produce time-intensity curves (TICs). A region of interest (ROI) of 1.6 cm (tubing diameter) was placed over the aorta of the phantom, and ROIs of 4 cm diameter were placed over the left and right myocardial sections, carefully including the complete section of the vessel and tissue compartment in the segmentation. Positioning of ROIs on the PET image plane corresponding to the MR image plane was determined from fusion of the dynamic 3D PET and 2D summed dynamic MR images using PMOD. ROIs were placed on PET images over the same spatial extent as the MR ROIs. The PET volumes of interest (VOIs) were 6.093 mm thick (3 PET slices) in the axial-dimension in order to match the slice thickness of the MR data (6 mm). All PET data were decay-corrected to the scan start time. We thus produced a set of TACs and TICs for the aorta and myocardial compartments over the range of *P*_*T*_.

### MR perfusion calculation

In-house software was used for perfusion quantification (Labview 2014 for Mac, National Instruments, Austin, USA). A model-independent deconvolution approach was used to calculate the tissue impulse response function, providing results in units of 1/s (Patel et al., 2010) and was not scaled to be in units of ml/g/min. Briefly, relative perfusion can be calculated based on the central volume principle using a signal deconvolution method (Jerosch-Herold et al., 1998). The TIC for the myocardial uptake function, *M(t),* can be calculated from the TIC for the arterial input function, *C*_*in*_ *(t)*, convolved with the tissue impulse response function *h(t)*:

1 $$ M(t)={\int}_0^t{C}_{in}\left(t\hbox{-} \tau \right)\cdot h(t) dt={\int}_0^t\left[{C}_{in}\left(\tau \right)\hbox{-} {C}_{out}\left(\tau \right)\right] d\tau $$

in which *C*_*out*_ *(t)* denotes the contrast concentrations in the venous out-perfusion. We performed this calculation using the pre-bolus curve, *C*_*in*_ *(t),* as an input function, in order to minimize the effect of signal saturation by the main bolus of higher GBCA concentration, an effect which has been noted in previous work in patients and with this phantom at high GBCA dosages (Chiribiri et al., 2013; Ishida et al., 2011). In the range of physiological concentration used in the pre-bolus injection, MR signal intensity is proportional to GBCA concentration. The tissue impulse response function *h(t)* has the shape of an exponential decay, and MR relative perfusion measurements were calculated from the *h(t)*) when *h(t = 0)*, i.e. at the peak value of the exponential decay. The delay between the arterial input TIC and the myocardial TIC was accounted for in the model (Zarinabad et al., 2013).

### PET perfusion calculation

PET data was modeled using a one-tissue compartment model characterized by a one blood compartment, one tissue compartment and two rate constants *K*_*1*_ (uptake rate constant in units of ml/g/min) and *k*_*2*_ (clearance rate from tissue to blood constant in units of min^−1^). For this phantom study, using [^18^F]F^−^ we assume an extraction fraction of 1.0 due to the lack of any metabolic processes, and thus the *K*_*1*_ constant is entirely representative of perfusion. In order to eliminate any prospective bias, PET and MR data were analyzed independently by two different authors blinded to the true myocardial perfusion rates, *P*_*T*_ (PET analysis by JOD, MR analysis by AC).

The terms ‘flow’ and ‘perfusion’ have been used interchangeably in both PET and MR literature. Owing to the fact that rates of liquid through our phantom were calibrated in terms of ml/g/min (i.e. units of perfusion) and *K*_*1*_ values from PET kinetic modeling were in the same units, we opt to keep consistency with terminology and use the term ‘perfusion’ rather than ‘flow’ (i.e. units of ml/min).

## Results

### Simultaneous imaging

**Fig. 2:**
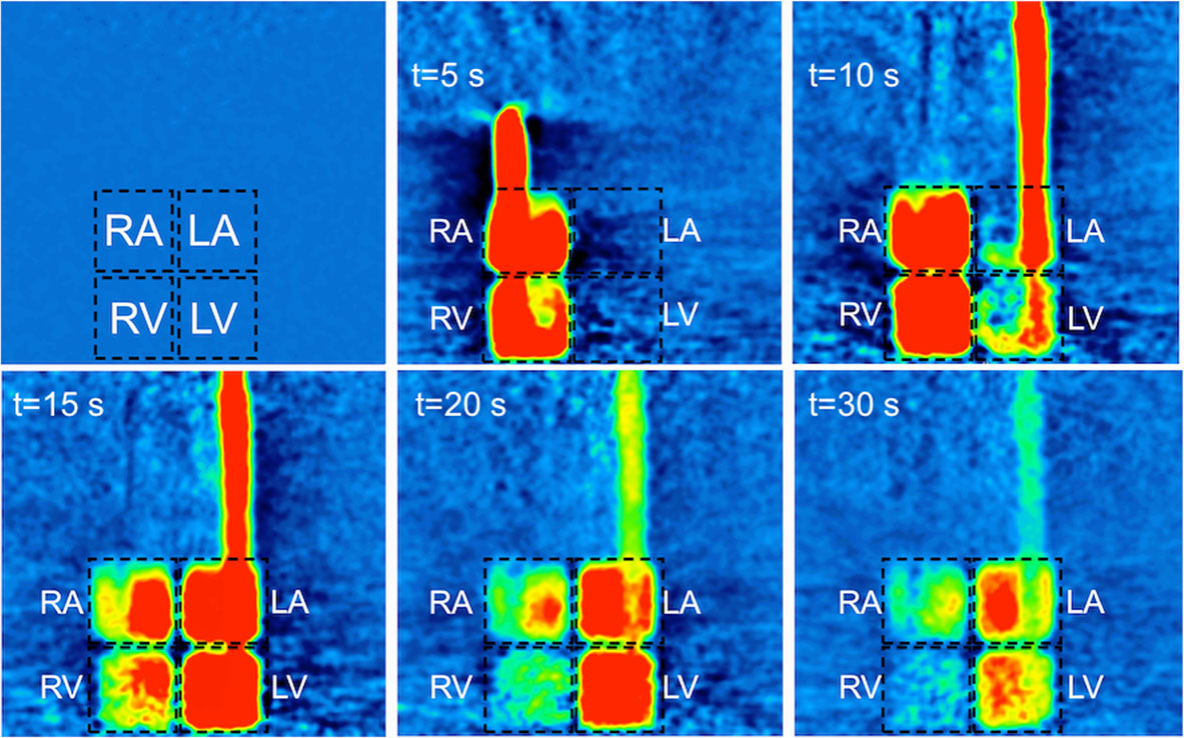
Single coronal PET slice from the 3D phantom acquisition at a cardiac output rate of 3 L/min, showing an example of radiotracer distribution in the myocardial chambers at increasing post-injection time points. All images are shown at the same windowing and level. RA/LA = right/left atrium, RV/LV-right/left ventricle

**Fig. 3:**
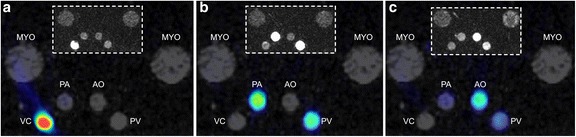
Example fused PET-MR images showing dynamics of GBCA and radiotracer transfer through the phantom. **a** – bolus in the VC (*t* = 0 s). **b** – outperfusion from the RV through the PA (*t* = 3 s), **c** – coronary circulation to the PV (*t* = 5 s) and the aorta (AO). Inset images show the time distribution of GBCA only

As the PET acquisition is fully 3D (25.8 cm field of view), all myocardial chambers can be visualized simultaneously. Figure 2 illustrates the rapid passage of radiotracer from the right atrium to ventricle, through the pulmonary circulation and into the left atrium and ventricle and exiting through the aorta. Figure 3 displays a fused transaxial image of the single MR slice with the corresponding merged 3 PET slices covering the same axial extent. The expected GBCA and PET radiotracer distribution through the phantom can be seen at increasing time points of the 2D MR imaging sequence and fused PET-MR images detailing the first pass dynamics of the phantom. The inset image of Fig. 3 shows the passage of MR contrast only, which temporally matches the distribution of PET radiotracer.

**Fig. 4:**
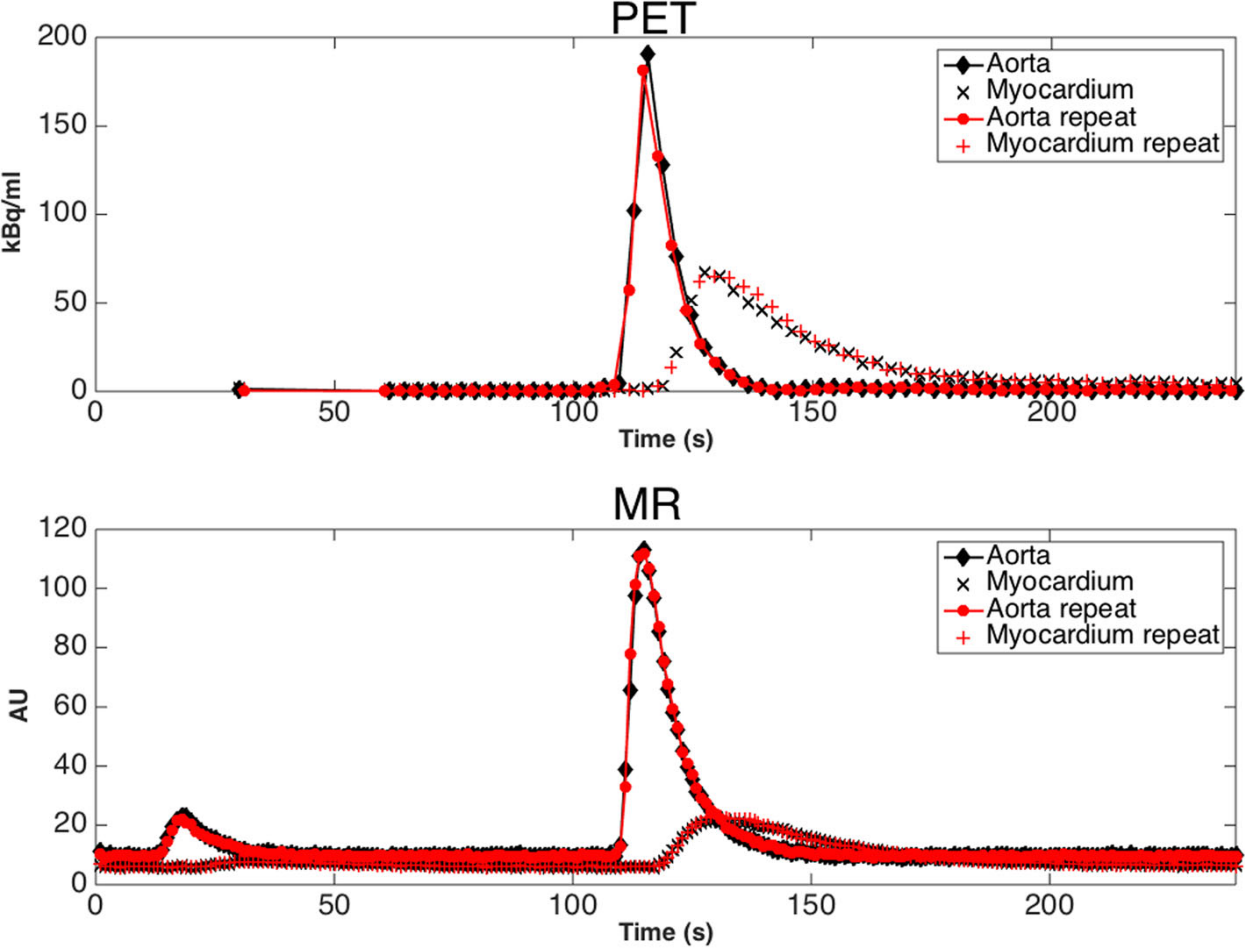
Comparison of mean activity concentration (kBq/ml) time activity curves (TAC) and mean MR signal (AU) time intensity curves (TIC) acquired from the phantom VOIs (PET-top) and ROIs (MR-bottom). Data are presented for simultaneous PET and MR acquisitions for a myocardial perfusion rate, *P*_*T*_ = 4 ml/g/min and cardiac output of 3 L/min. Repeat scan data using ROI and VOI in the same positions are also plotted and show a high level of repeatability. Error bars are omitted in order to improve visual clarity of overlapping traces

**Fig. 5:**
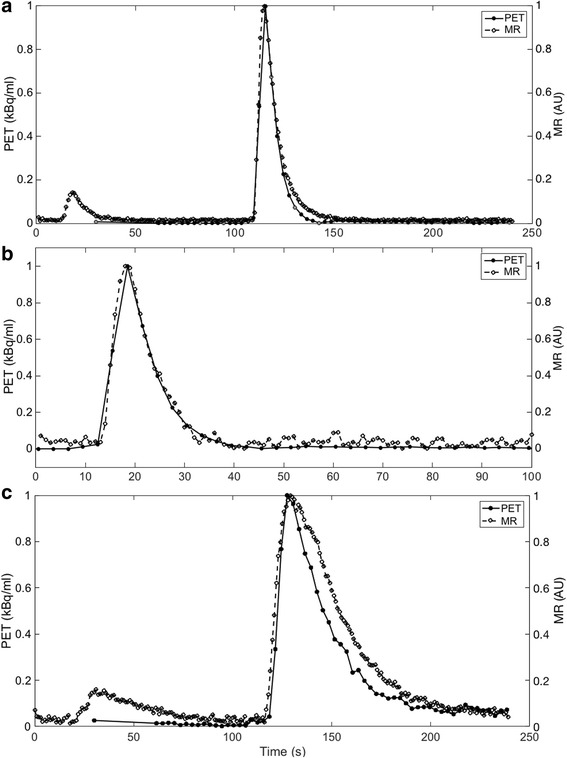
Comparison of normalized (respective maximum signal intensity) AIFs derived from both PET and MR signal traces at a myocardial perfusion rate of 4 ml/g/min and cardiac output of 3 l/min. Image (**a**) represents the input functions from the main MR bolus and PET bolus, showing a longer washout of GBCA than radiotracer in the main bolus. Image (**b**) details the MR prebolus with the same PET bolus as (**a**) but time-shifted to provide comparison. Image (**c**) details the simultaneous curves from the myocardial compartment showing a clear difference in transit time, potentially due to higher mass and viscosity of the GBCA

Image processing of the ROI/VOIs to produce TACs and TICs allows comparison of resulting mean PET kBq/ml to MR signal intensity during transit of the GBCA and radiotracer, as shown in Fig. 4. As the repeat injection of [^18^F]F^−^ and GBCA was performed using the same timings and methodology as the first test, the time traces produced by both PET and MR data were similar. Although not shown in Fig. 4 for clarity, for a single *P*_*T*_ of 4 ml/g/min, one standard deviation of the mean PET activity concentration from the VOI varied over time from ±2 to ±24%, while that of the MR mean ROI signal intensity varied over time from ±13% to ±29%. Standard deviations were similar for other values of *P*_*T*_. Figure 5 shows a comparison of the input functions from both imaging methodologies normalised by their respective maximum signal intensities, firstly between the main MR bolus peak of GBCA and the radiotracer (A), secondly between the MR prebolus peak (which was used for the MR perfusion analysis) and a time-shifted PET TAC overlaid to provide comparison (B), and finally a normalized comparison of the functions obtained from the myocardial chamber (C). From these traces, it can be observed that the input functions for both PET and MR models show similar characteristics.

### Perfusion calculations

**Fig. 6:**
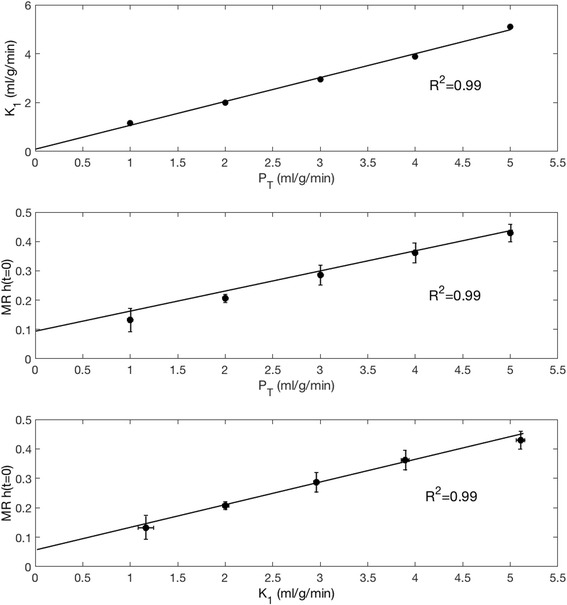
Top - Resulting *K*_*1*_ values from a single compartment model for PET data plotted against *P*_*T*_. Middle – MR values of *h(t = 0)* from model-independent deconvolution for MR images plotted against the range of *P*_*T*_. Bottom - MR values of *h(t = 0)* plotted against the range of *K*_*1*_ from PET kinetic modeling

PET datasets were used to calculate perfusion (ml/g/min) via *K*_*1*_, and MR datasets to calculate relative perfusion values via *h(t = 0)* as described above. Resulting *K*_*1*_ and *h(t = 0)* and are shown in Table 1. Figure 6 shows three plots detailing the relationship between *K*_*1*_ and *P*_*T*_, *h(t = 0)* and *P*_*T*_ and also *h(t = 0)* and *K*_*1*_. The results show that *K*_*1*_ is linearly related to *P*_*T*_ (R^2^ = 0.99), and that *h(t = 0)* is also linearly related to both *P*_*T*_ and *K*_*1*_ (*R* ^2^ values of 0.99 in both cases).

**Table 1:** Results of employing a one compartment kinetic model to the PET and deconvolution model to the MR data (Eq. 1)

	PET				MR	
*P*_*T*_	*K*_*1*_ (ml/g/min)	*K*_*1*_ SE (%)	k_2_ (l/min)	*k*_*2*_ SE (%)	*h(t = 0)* (s^−1^)	*h(t = 0)* SE (%)
(ml/g/min)						
1	1.14	3.8	0.96	7.83	0.132	4.019
2	1.93	2	1.62	1.71	0.213	4.141
3	2.94	1.55	2.44	2.05	0.279	3.346
4	3.81	2.41	3.20	2.93	0.339	3.834
5	5.14	1.83	4.43	3.35	0.403	2.985
REPEAT						
1	1.18	0.5	0.99	7.68	0.133	3.953
2	2.07	1.87	1.87	1.74	0.201	3.112
3	2.98	2.6	2.60	2.06	0.293	4.526
4	3.98	3.21	3.21	2.16	0.384	4.259
5	5.07	4.44	4.43	3.48	0.457	3.214

## Discussion

We performed PET-MR tests using a specialized cardiac phantom allowing assessment of myocardial perfusion measurements with both imaging methodologies from simultaneously acquired data. Both PET and MR are accurate tools for the assessment of myocardial ischaemia, however there are drawbacks to each technique. For example in MR, derivation of fully quantitative perfusion units remain a complex process due to the relationship between signal intensity and gadolinium contrast and dependence on acquisition sequence (Jerosch-Herold, 2010), Also 2D imaging is favoured due to dynamic imaging meaning that perfusion calculations cannot be representative of the entire volume. In PET, the cost of the perfusion exam can be prohibitive and the procedure is based on access to short-lived radiotracers thus requiring access to a cyclotron. There is also a not insignificant radiation dose associated with the radiotracer. Recent work has investigated the complimentary information generated by simultaneous late gadolinium enhancement and 18F–FDG imaging (Rischpler et al., 2015). Although the principles of image formation between MR and PET are based entirely on different physical principles, we have shown that similar TACs and TICs from the arterial and myocardial compartments can be obtained from a single short acquisition. Furthermore, it has been possible to show that the results obtained with the employed MR and PET models are linearly related to the true myocardial perfusion rate, *P*_*T*_.

The phantom is physiologically relevant and as such is able to explore some relevant aspects of perfusion dynamics of the human heart. Figures 2 and 3 demonstrate the distribution of both GBCA and radiotracer through the right side of the cardiac chambers followed by the left, with perfusion through the myocardial compartments following shortly after. Simultaneous traces of PET activity concentration and MR signal intensity in Fig. 4 demonstrate the transit of radiotracer and GBCA through the phantom, showing that true simultaneity of PET and MR signals can be achieved in this phantom. Our data also indicate that when a dual bolus approach is used in MR, linear perfusion estimates to those obtained in PET can be achieved.

In this study, we adopted a dual-bolus injection scheme previously described and validated by our group (Ishida et al., 2011; Schuster et al., 2013). The results of this study demonstrate that this approach results in MR input functions which are very similar in shape and transit time to the reference standard PET input functions (Fig. 5). The myocardial compartment TAC and TIC closely match in terms of wash-in, however the MR TIC can be observed to have a longer transit time than the radiotracer (Fig. 5, part C). We propose that this may be due to the higher particle mass and viscosity of the GBCA than the radiotracer.

One of the main benefits of the phantom model is its reproducibility. Figure 4 demonstrates that repeat acquisitions at the same *P*_*T*_ give similar TAC and TIC. Upon calculation of perfusion via PET (Table 1), *K*_*1*_ values when performed with independent repeat acquisitions produce values in the range of 1.2% to 7.5% of each other. A similar repeatability is shown in MR data with a repeatability of 0.6%–13% for *h(t = 0)*. PET measurements at *P*_*T*_ = 1 ml/g/min showed an overestimation of *P*_*T*_ by 16%, however the *P*_*T*_ rates of 2, 3, 4 and 5 ml/g/min were accurate to *K*_*1*_ values to within a maximum of 2.65%, indicating good precision for repeated measurements and also a good accuracy to *P*_*T*_ values above 1 ml/g/min. Inaccuracies in the true measurement of *P*_*T*_ = 1 ml/g/min due to physical accuracy of the roller pumps may account for the larger differences at this value of *P*_*T*_, and will be investigated in the next generation of the phantom currently under development. Although *h(t = 0)* values represent relative perfusion measurement and were not scaled to represent absolute perfusion units, their relationship to *P*_*T*_ and *K*_*1*_ can be clearly observed in Fig. 6, whereby a linear relationship was found between *h(t = 0)* and *K*_*1*_, as well as *h(t = 0)* and *P*_*T*_. Another potential advantage of the approach is the possibility to address differences in the way images are acquired and modality-specific artifacts, such as saturation effects in MR or attenuation correction in PET.

Total analysis time of each series of PET images was approximately 30 min, and MR images were approximately 5–10 min. Owing to the geometrical differences between phantom and patient images, semi-automated PET analysis software could not be used. Furthermore, PET images were rebinned into short frames of 3 s because of the rapid transit of the radiotracer in water. In clinical image we expect a lower amount of data and frames to analyse.

Attenuation correction is a major issue in clinical PET-MR imaging and the focus of much research (Mehranian & Zaidi, 2015). In this work, we utilised GBCA for the bolus injection, and in clinical studies the concentration used would be far higher. Previous work by our group has shown that despite large concentrations of GBCA up to 65 mM (presenting the scenario of GBCA bolus in the left ventricle simultaneously with the PET radiotracer), the effect of attenuation of gamma photons by GBCA on quantified activity concentration (kBq/ml) in the final reconstructed images is less than 5% when compared to no GBCA present (O'Doherty & Schleyer, 2017).

We believe that by providing a standardized setup and known perfusion rates, results, claims and hypotheses from clinical studies can be further investigated. For example, in a recent study by our group, MFR of 41 patients calculated from independent CMR and PET scans have been shown to correlate well, however absolute CMR perfusion at stress and rest correlated weakly and were positively biased compared to their PET counterparts (Morton et al., 2012). This may indicate that errors in quantification have a similar effect on stress and rest perfusion MBF values but are cancelled by calculation of the MFR. Future experiments could verify this finding by the exclusion of physiological variation. The phantom also allows comparison of kinetic models given the known ground truth of perfusion rates, and the potential for development of new hybrid kinetic models employing both PET and MR data.

Knowledge of the relationship between *P*_*T*_, *K*_*1*_ and *h(t = 0)* may allow the creation of a modality-specific calibration curves. Particularly in the case of perfusion MR, this could allow converting the results of the deconvolution operation from seconds^−1^ to ml/g/min of perfusion. This approach could prove of value as a substitute for current approaches based on constraining the deconvolution operation (Zarinabad et al., 2013; Hautvast et al., 2012). This may lead to an improvement in the correlation between absolute MBF values measured with MR and PET, which was shown to be suboptimal in comparison with MPR values in previous studies (Morton et al., 2012).

### Limitations

The use of the phantom in this work for simultaneous PET-MR acquisitions as a surrogate for clinical acquisitions presents some fundamental limitations. Despite the fact that the TIC and TAC curves are similar in appearance for this simplified phantom study, it may not be the case for clinical studies in a human cohort due to the mechanism of transport of radiotracer (intracellular) and GBCA (extracellular). Therefore the phantom study serves to provide preliminary investigation into the standardized comparison between PET and MR perfusion values in a controlled simulation. The phantom model used in our experiments is not able to capture the broad range of body structures and physiological states that may be present in a clinical setting and as such represents an oversimplification of the cardiovascular system which cannot detail true myocardial diffusion or radiotracer uptake. Furthermore, despite a good correlation between PET and MR perfusion, there remain fundamental differences between the calculation methodology between the MR model-independent deconvolution approach (leading to a parameter related to perfusion) and perfusion as calculated from a single compartment PET model. As is the mechanism with PET radiotracers, no separate tissue compartment exists, for example one with well-defined mechanical properties such as a membrane. Therefore true intracellular uptake cannot be simulated, only allowing non-circulating extravascular transfer of tracer. Strategies would be required in order to simulate the kinetics of perfusion tracers that undergo metabolic processes such as [^13^N]NH_3_ or [^18^F]flurpiridaz. Efforts should be made to create myocardial compartments within the phantom, which would allow a more accurate approach to kinetic modeling. Furthermore, we performed only 1 repeat acquisition of each *P*_*T*_ in this feasibility study, further repeat measurements would allow the calculation of a repeatability coefficient for both the PET and MR datasets.

The current phantom model setup is unable to reproduce the multiple sources of image artifacts in PET-MR such as the effects of motion due to respiratory or cardiac contraction. Thus the phantom allows an environment free from these potentially confounding effects focusing only on the assessment of the perfusion dynamics within the cardiac compartments. However, translation of calibrations from the phantom to the clinical setting should be treated with caution. Confounding factors from clinical data may include the use of respiratory correction via importing an average cine CT or using MR-based navigators (Ouyang et al., 2013) or employing MR motion-field based cardiac motion correction employed in PET reconstruction (Huang et al., 2015). Efforts to apply these techniques specifically to quantitative dynamic PET-MR cardiology are in their infancy, although some techniques are currently under development for static imaging (Nensa et al., 2013; Vontobel et al., 2015).

## Conclusion

We have performed a feasibility study of the first simultaneous PET-MR acquisitions from a dynamic cardiac perfusion phantom, showing similar first-pass dynamics of both the PET and MR contrast agents. We have described the resulting simultaneous traces, showed initial repeatability of the phantom studies and also demonstrated a correlation between perfusion quantification of the PET time-activity traces using a kinetic model, relative MR perfusion using a deconvolution model and the true manually set myocardial perfusion rate. The phantom shows potential for improving standardisation of perfusion measurements, analysis routines, development of imaging protocols and potential calibration of MR perfusion values. We have also described the major limitations of the system, detailing how these phantom studies are an important stepping stone allowing investigation of sequence development/comparison and kinetic model development in both PET and MR modalities.

## Abbreviations

CAD: Coronary artery disease
CMR: Cardiac magnetic resonance
GBCA: Gadolinium based contrast agent
MFR: Myocardial flow reserve
MPI: Myocardial perfusion imaging
PET-MR: Positron emission tomography & magnetic resonance
